# History of the Royal Medical Society for the Year 1779; with the Memoirs Relative to Physic and Medical Philosophy for the Same Year, Taken from the Registers of the Society

**Published:** 1784

**Authors:** 


THE
LONDON MEDICAL JOURNAL,
For APRIL, MAY, and JUNE,
1784.

### SECTION I.

### BOOKS.

I. *Hiſtoire de la Société Royale de Medecine. Année* 1779. *Avec les Memoires de Medecine et de Phyſique Medicale pour la meme année, tirés des Regiſtres de cette Société*, i. e. *Hiſtory of the Royal Medical Society for the year* 1779; *with the Memoirs relative to Phyſic and Medical Philoſophy for the ſame year, taken from the Regiſters of the Society.* 4to. Paris, 1782. 958 pages, with 15 copper-plates.

IN the hiſtorical part of this volume, we have an account of the prizes diſtributed by the Society ſince the publication of their ſecond volume; and a copy of the king's edict which inveſts them with the direction of all the mineral waters in the French dominions, and the privilege of examining, approving, or profcribing all quack medicines. After thefe follow the eulogies of the following deceafed affociates; viz. Meffieurs Le Roy, Navier, Bucquet, Lieutaud, and Gaubius; and likewife fhort accounts (under the title of *Notices*) of the late Meffieurs Bonafos and Bernard, affociates, and M. Planchon, a correfpondent, of the Society.—We fhall review each of thefe in the order in which they ftand.

Charles le Roy was born at Paris, January the 12th, 1726. He was the youngeft of four fons of Julian le Roy, a famous watch-maker, and ftudied phyfic firft at Paris and afterwards at Montpellier, where he graduated in 1752. But before this; viz. in 1750, he travelled into Italy, and at his return, in 1751, communicated to the Academy of Sciences at Paris fome obfervations, made in his travels, on the *Grotto del Cani*, and the luminous appearance of the fea. His experiments on the dew foon after procured him the honour of being elected of the Academies of Sciences at Montpellier and Paris, and of the Royal Society of London.——In 1759, he was appointed to a Medical Profefforfhip at Montpellier, where he obtained confiderable reputation as a teacher and practitioner titioner of physic, and likewise as a medical writer. He devoted much of his leisure time to chemical enquiries, and after the death of M. Venel was appointed by government to revise and complete the work undertaken by that celebrated chemist on the mineral waters of France. The materials of this work are now in the possession of the Society.——In 1777, on the death of M. de Bordeu he was induced to remove from Montpellier to the capital, where his professional talents were known and esteemed. Instead of purchasing, as is usually done, a medical appointment, which might give him the privilege of practising at Paris, he chose rather to go through the forms prescribed by the College of Physicians, being of opinion, as he observed to his friends on this occasion, that a physician cannot possess too many claims to the confidence of the public. He had not been many months, however, in this new situation before he began to vomit after meals, and to have lancinating pains and other symptoms of a schirrous pylorus, which proved fatal to him on the 10th of December, 1779. —Of his medical writings the most interesting is his work on the Prognostic in acute diseases, of which an Engliſh tranſlation has lately been publiſhed (ſee our 3d vol. p. 257).

Peter Toussaint Navier was born Nov. 1, 1712, at St. Dizier in Champagne, and after graduating at Rheims, in 1741, took up his reſidence at Chalons ſur Marne, where he afterwards became one of the inſtitutors of an Academy of Sciences, to which he communicated many uſeful papers; but the work which gained him the moſt reputation was on the antidotes to arſenic and other poiſons (*Contrepoiſons de l'Arſenic*, &c.) in 2 vols. 8vo. Publiſhed in 1778. He died at Chalons, July 16, 1779, aged 67 years.

John Baptist Michael Bucquet was born at Paris, Feb. 18, 1746. His father, who was an advocate, deſigned him for his own profeſſion; but he ſoon quitted the ſtudy of the law and purſued that of phyſic. He was one of the firſt members of the Royal Medical Society, and in 1777 he was appointed by the College of Phyſicians to ſucceed M. Roux as profeſſor of chemiſtry. He likewiſe gave private lectures on chemiſtry and natural hiſtory. His knowledge and eloquence eminently qualified him for the office of a teacher; but frequent attacks of a moſt painful colic interrupted his purſuits, and at at length terminated in death in Jan. 1780, before he had compleated his 34th year. During the laſt courſe of his lectures he uſed to go from his bed to his lecture-room, and there his moans ceaſed, and he forced a placid countenance. During his lecture, when his pains were exceſſive, he preſſed againſt the table, but never loſt ſight of his ſubject. His pupils liſtened to him with pity and aſtoniſhment. After the courſe was ended he determined to indulge in the uſe of ſedatives. Of æther, which had conſtantly afforded him a temporary relief, he at laſt ſwallowed a Paris pint every day, taking alſo in the ſame ſpace of time an hundred grains of opium. After his death the principal diſeaſe was found to be in the colon, which was contracted, ſchirrous, and ulcerated. The ſtomach and inteſtines were inflamed and ſoftened by the uſe of æther, and the bile in the gall-bladder was of a roſe colour.—He was author of ſeveral papers publiſhed by the Society, and by the Academy of Sciences, of which he was alſo a member.

Joseph Lieutaud was the ſon of an advocate, and was born at Aix, June 21, 1703. The relation, who ſuperintended his education, was named Garidel (not Lieutaud, as we formerly merly mentioned, vol. i. p. 279) being his uncle by his mother's ſide. In the caſe of the duke of Burgundy, when the phyſicians and ſurgeons, who were conſulted, differed in opinion, they were ordered to deliver their ſentiments in writing, M. Lieutaud, who was one of the number, wrote, "the complaint of his "highneſs is a luxation of the thigh, occaſion- "ed by a contuſion of the cartilages, liga- "ments, and ſynovial glands of the cotyloid "cavity." This opinion was afterwards found to have been well founded.

After the death of M. de Senac, the place of Firſt Phyſician was for ſome time put in commiſſion; but when the preſent king of France came to the throne he veſted it in M. Lieutaud. His majeſty, who was fond of converſing with him, one day aſked him about a phyſician, who had been exceſſively praiſed in his preſence. "Sire," ſaid M. Lieutaud, "this man "is very far from deſerving any of the fine "things that have been ſaid of him; but it is "often in this manner that the great pay their "phyſicians."

He died after a ſhort illneſs. There remain of his family a ſiſter, who is in her eighty-ſixth year, year, a brother who is a Francifcan monk, and feveral nephews.

JEROM DAVID GAUBIUS was born at Heidelberg, Feb. 24, 1705. His grandfather was a colonel of horfe, who died leaving four fons to fubfift on a fmall penfion, which they loft at the death of the elector palatine Charles. Chriftopher, the youngeft, who was the father of our phyfician, engaged in trade at Heidelberg, and though a proteftant, placed his fon at the Jefuits college in that city; but fearing they might make him a profelyte to their religion, he removed him to the Orphan-houfe at Halle, where his mafter is faid to have treated him harfhly, and to have confidered him as a youth deftitute of genius. But Gaubius perfifting in his attachment to a learned profeffion in preference to trade, for which his father had defigned him, was fent to Amfterdam to be under the care of his uncle, John Gaubius, who practifed with great reputation in that city as a phyfician for fifty years, and is known by an *Epiftola problematica* in Ruyfch's works. By this relation, who firft added an *us* to the family name of Gaube, young Gaubius was fent firft to Harderwyk, and afterwards to Leyden, where he attracted the notice of Boerhaave, and took the degree of doctor tor of phyſic in 1725. After quitting Leyden he paſſed a year at Paris, and went from thence to Straſburgh and Heidelberg, where his ſtay was but ſhort, as he ſoon returned to his uncle at Amſterdam, whoſe daughter he afterwards married. By this marriage he had ſix children, of whom only one daughter ſurvives him. Soon after his return to Amſterdam he was appointed phyſician to the town of Deventer, where he went to reſide after having conſulted Boerhaave, who promiſed never to loſe ſight of him in whatever country he might chance to fix. At Deventer he applied himſelf much to the ſtudy of pharmacy. In 1731, Boerhaave beginning to feel the infirmities of old age reſigned his botanic chair to Van Royen, and his chemical profeſſorſhip to Gaubius. In 1734 he was elected profeſſor of phyſic, and then became the colleague of his maſter, who died four years after.

The writings of Gaubius are few in number, but they have eſtabliſhed his reputation on the moſt ſolid foundation. His *Inſtitutiones pathologiæ medicinalis*, *Adverſaria varii argumenti*, and his diſcourſes *de Regimine mentis*, are all admired for the elegant ſtyle in which they are written, and for the clear and accurate views they exhibit of the different ſubjects on which they treat.—

In

In a paper in the Harlem Memoirs he gives an account of a caſe of inoculation of the ſmall pox which had like to have ended fatally, and which afterwards made him not very friendly to that practice.

His medical fame ſpread throughout Europe, and the empreſs Elizabeth invited him to St. Peterſburgh to be her firſt phyſician, but he declined accepting this flattering offer. In 1761 the States General appointed him firſt phyſician to the ſtadtholder, then a minor; and in 1768, when the prince came of age and honoured the univerſity of Leyden with his preſence, Gaubius compoſed ſome Latin verſes on the occaſion, which were much applauded.—He preſerved his health till the age of ſeventy, when he began to be troubled with the gout. In November 1780, after returning from the Hague, he was attacked with a fever, which carried him off on the 29th day of the ſame month, in his 75th year. He has left a large fortune to his daughter, Maria Amelia, who is married to Mr. Henry Twent, one of the magiſtrates of Leyden.

Joseph Bonafos, ſenior member of the faculty of phyſic at Perpignan, was born there December 4, 1725. He was phyſician to the Military Hoſpital and Hotel-Dieu in that city, and died on the 5th of February 1779 of a pu-

 trid trid fever. He has left behind him a MS. treatise on the Practice of Physic.

John Bernard was born May 14, 1702, at Nantes, where his father was a physician. He graduated at Montpellier, and practised first at Rochelle; but M. d'Aguessau, chancellor of France, who patronized him, procured for him a medical professorship at Douay, where he taught anatomy and physiology with much reputation. His physiological doctrines are contained in seven dissertations published at different times in Latin. He was elected of different academies, and among others of the Royal Society of London. He was a man of pleasantry, and even in scholastic ceremonies found it difficult to preserve a grave countenance, but he is said to have been strict in his examination of candidates for academical degrees. He died of a strangulated hernia in 1781, aged 80 years.

John Baptist Luke Planchon was born at Renaix in Flanders, Nov. 5, 1734. His father, who was a physician, practised at Leuze, a small town in Hainault, and was ruined by a fire, which destroyed the greatest part of the town in 1742, so that he was incapable of giving his son a learned education. For this he was indebted debted to his uncle, who was in the church, and ſent him firſt to ſtudy at Ath, and afterwards at Louvaine, where he was admitted a licentiate in phyſic in 1758. He ſettled as a phyſician at Tournay, and was the author of ſome ingenious diſſertations which obtained the prizes offered by different academies, and among others of a treatiſe on the miliary fever of lying-in women, which obtained the approbation of the faculty of phyſic at Paris in 1778. The late empreſs of Germany honoured him with a gold medal, and ordered Baron Störck her phyſician to thank him for his ſervices during an epidemic diſeaſe that had prevailed in the Low Countries. He died of a fever Nov. 6, 1781, aged 47 years.

*Meteorological Obſervations, by Father Cotte.*— Theſe are on the ſame plan as thoſe of the former volume. (See our 4th vol. p. 41.)

*Practice of Phyſic.*—1. *Obſervations on the venereal diſeaſe, and the thruſh with which new-born children are attacked, with reflections on the nature and treatment of theſe two diſorders.* By M. Colombier.—An hoſpital called *Hoſpice de Vaugirard*, has lately been eſtabliſhed near Paris for the reception and cure of infants afflicted with the venereal diſeaſe, and likewiſe of their mothers or nurſes. The greater number of ſuch infected

 chilchildren, we are told, are born before the ufual period. The firft circumftance that ftrikes the phyfician on feeing them is, that the fkin of the face as well as of the reft of the body, is fhrivelled, and that they feem to bear the marks of old age. Many of thefe poor children are reduced to a true ftate of marafmus, and in the advanced ftage of the difeafe the lips, tongue, and fauces are covered with chancrous apthæ, which are found to extend along the larynx, and even down the pharynx and œfophagus to the internal furface of the ftomach. In fome the armpits, nipples, and belly are ulcerated; but the moft common fymptom is an ophthalmia, which in the flighter ftate of the difeafe is confined to the eye-lids, but in the more advanced ftage affects the tunica conjunctiva. In both cafes the great angle of the eye is filled with purulent matter, and fometimes is ulcerated. In fome patients the parts of generation are affected, and almoft all have a diarrhœa. In thefe cafes mercurials, though adminiftered in fmall dofes, having been found to be too active, our author was induced to try the effect of mercurial frictions on the mothers and nurfes of the patients. For this purpofe a number of pregnant women afflicted with lues, were admitted into the the hoſpital to lie-in and ſuckle their children, on condition that each of them, if their ſtrength would permit, ſhould ſuckle another infected child. Infected nurſes were likewiſe admitted on the ſame condition. The cure was effected by bathing and frictions as in the Montpellier method, or cure by extinction as it is called. From June 1780 to July 1781, 136 infected children were admitted into the hoſpital. Of this number 42 were cured of their venereal complaints; the remainder died. Concerning the latter, however, we are told, that many of them were ſo much diſeaſed, when brought to the hoſpital, that they died in a day or two after their admiſſion. Our author likewiſe informs us, that from calculations it appears, that in this hoſpital a greater proportion of infected children are ſaved than of healthy children intruſted to the care of nurſes, and that before this inſtitution was ſet on foot hardly any children were preſerved when infected with the venereal diſeaſe.

The thruſh (which our author diſtinguiſhes by the names of *Millet* and *Muguet*, neither of which are to be met with in the writings of Sauvages) is another diſeaſe that particularly affects new-born children, and under ſome circumſtances cumſtances becomes contagious. The ſymptoms of it are ſmall, white, hard puſtules on the lips, tongue, and even alimentary canal, which prevent deglutition. Theſe are ſucceeded by diarrhoea; the face grows pale, and at length purple ſpots appear on the body, and may be conſidered as the ſigns of death. This diſeaſe, when contagious, is aſcribed by our author to the noxious effluvia produced by crowding together a great number of children in confined apartments. Nine children, we are told, who had remained only twenty-four hours in a room where it prevailed, were all of them attacked with it. A pure air and wholeſome diet, cleanlineſs, mild tonics, and expoſure to the vapour of mercury for a few hours every day, are ſaid to be the only means of cure as yet diſcovered. Care is to be taken to purify the apartments by freſh air and other means.—2. *New Obſervations on Medical Electricity*, by M. Mauduyt.—3. *Account of a Solvent of biliary Concretions*, by M. Durande. This ſolvent had been ſpoken of in a former volume (ſee our 3d vol. p. 160); but no mention had been made of the proportions of the ingredients that compoſe it, or the method of adminiſtering it. We are now directed to mix two drachms of ſpirit of turpentine, pentine, with three drachms of æther, in a Florence flaſk well ſtopped. A fifth or even a fourth part of this mixture is to be ſwallowed every morning; but, before recourſe is had to ſo heating a remedy, cooling remedies, ſuch as venæſection, warm bathing, and a milk diet are to be employed. After each doſe of the ſolvent the patient is to ſwallow ſome veal broth. M. Durande thinks it right to wait till the concretions are diſſolved before any purgative medicines are adminiſtered, as without this precaution a violent colic is ſometimes produced; but he has omitted to inform us by what ſigns we may learn that the ſolvent has produced the deſired effect. In ſome caſes, we are told, the liver has been rendered painful, and violent colic excited, by the uſe of this remedy; but theſe ſymptoms have eaſily given way to the uſe of aſſes milk, mucilaginous drinks, &c.—4. *Reſearches and Obſervations on various Subjects of Phyſic, Surgery, and Anatomy*, by M. Vicq d'Azyr. In this paper the author has combined ſeveral communications of the Society's correſpondents, with others preſented by himſelf. He firſt treats of concretions of the ſtomach and inteſtines, concretions of the kidnies, urinary bladder, uterus, and gall-bladder.

Some

Some obſervations on diſeaſes of the bones likewiſe make part of the paper, and among other things we have an account of an anchyloſis of the os humeri with the ſcapula in a ſcrophulous ſubject. Next follow remarks on the treatment of aneuriſmal tumours by compreſſion, and a deſcription of the diſpoſition of the veſſels in the pituitary membrane of the horſe and ruminating animals. The paper cloſes with an account of the ſtate of the bodies in the vault of the church belonging to the Cordeliers at Toulouſe. This church was built about the middle of the 16th century, and there are bodies in the vault under it which have been there ſince that time, and were ſeen by the Editor of this Journal in 1776. They have long been an object of curioſity to travellers. M. Vicq d'Azyr, having brought ſeveral limbs of theſe bodies to Paris, found their levity very great. The bodies themſelves commonly weigh from nine to twelve pounds each. There remains only the baſis of the parts that had the moſt conſiſtence, and when the dry, tanned-like ſkin is removed, inſects are found in the place of cellular membrane.——The different ſubjects deſcribed in this paper are illuſtrated by a great number of elegant figures in nine plates.

[*To be continued.*]

